# Determination of nurses’ happiness, hope, future expectations, and the factors influencing them: a descriptive study that can guide policy development to prevent nurse migration*

**DOI:** 10.1186/s12912-024-01876-2

**Published:** 2024-03-26

**Authors:** Derya Yanık, Çiçek Ediz

**Affiliations:** 1 Nursing Department, Faculty of Health Sciences, Batman University, 72060 Batman, Turkey; 2 Department of Psychiatric Nursing, Faculty of Health Sciences, Hakkari University, Hakkari, Turkey

**Keywords:** Future expectation, Happiness, Hope, Income, Nurses, Migrating

## Abstract

**Background:**

The happiness and hopefulness of nurses are not only known that contribute to their emotional well-being but also professional creativity, improve the quality of nursing services and organizational performance. Therefore, knowing which factors affect nurses’ mental well-being and future expectations can provide information for nursing workforce planning policies. This study was conducted to determination of Nurses’ happiness, hope, and future expectations and the factors influencing them.

**Method:**

326 nurses from 52 provinces of Turkey participated in this descriptive study. The data collection instruments included a Demographic Characteristics Form and questions from the Turkish Statistical Institute’s Life Satisfaction Survey to assess overall happiness, hope, and expectation levels. The study data was collected using an e-survey prepared through Google Forms in line with the principle of voluntarism. In the study adhered to the EQUATOR checklist for descriptive studies.

**Results:**

The average scores for overall happiness, hope, and future expectations among the participating nurses were found to be 2.34 ± 0.98, 2.22 ± 0.95, and 1.26 ± 0.54, respectively. It was determined that the levels of happiness, hope, and future expectations of nurses are influenced by satisfaction with income, income’s ability to meet needs, and personal development over the last five years.

**Conclusion:**

The study concluded that the overall happiness and hope levels of nurses are low, while their future expectations are at a moderate level. Satisfaction with income affects the happiness and hope levels of nurses. Three quarters of the nurses participating in the study want to work abroad. This situation may lead to a need for qualified nurses in the future.

## Introduction

Positive psychology, which focuses on individuals’ personal experiences and emphasizes their strengths rather than weaknesses, highlights concepts such as satisfaction and well-being for the past, happiness for the present, and hope and expectations for the future [1, 2]. The concept of happiness, commonly used as an indicator of individuals’ subjective well-being, is an individual phenomenon that involves how one perceives and evaluates their own life, expressing life satisfaction and a positive mental state [3, 4]. Happiness, widely regarded as a significant source of motivation in human life, signifies experiencing positive emotions more frequently, deriving pleasure from life, and having a high level of well-being [5–7]. Throughout individuals’ lives, happiness is a sought-after goal and often considered a purpose of life, intertwined with various factors such as personal characteristics, social relationships, religious beliefs, socio-demographic attributes, law, democracy, economic, cultural, and political elements [3, 4, 8].

Another significant source of motivation essential for enhancing individuals’ well-being and maintaining their mental health is hope. Hope is considered a form of energy and a pathway toward goal-oriented behavior that focuses on internal feelings of achievement, motivates action, and sustains life [9, 10]. Low levels of hope can lead to feelings of distress, while high levels of hope can lead individuals to experience positive emotions and thoughts about the future [11, 12]. In this context, hope not only influences an individual’s mental well-being but also shapes their expectations for the future. *Expectation* refers to the process of making inferences about the future based on past life experiences and shapes an individual’s plans, interests, and concerns for the future [13]. The expectation for the future, which forms the core belief about the future, contributes to an increase in hope among individuals with a positive future orientation, while individuals with negative future expectations may experience hindered behaviors and a sense of hopelessness [14].

The high level of happiness and hope contributes to the increase in an individual’s future expectations. Consequently, individuals with a high level of subjective well-being tend to be more efficient, productive, and innovative in both their family and social lives as well as their professional careers [15]. Subjective well-being is important in all professions, but especially the nursing profession, where the self is used therapeutically, is directly related to the sense of well-being [16]. The happiness and hopefulness of nurses are not only believed to contribute to their emotional well-being but also enhance professional creativity, improve the quality of nursing services, reduce intentions to leave the job and feelings of burnout, and increase organizational performance [10, 17–20].

Nurses’ decisions to migrate to more developed countries are influenced by their levels of hope and future expectations [21]. In certain developing countries, migration serves as a pathway to financial security and social mobility and is often regarded as a cultural aspiration. Factors such as national and global inequalities, as well as ongoing development and underdevelopment processes, play significant roles in shaping migration behaviors [21, 22]. The phenomenon of brain drain within health services has become a pressing issue in Turkey, mirroring global concerns [23]. Turkey has faced economic challenges, with an inflation rate exceeding 50% since 2022, according to Turkish Statistical Institute (TUIK) [24]. Despite this, public service salaries have not kept pace with inflation, leading to a gradual decrease in the purchasing power of nurses. The pursuit of higher income and improved prosperity is identified as one of the driving forces behind the decision to migrate abroad [22]. Compounding these challenges, Turkey experiences a growing disparity between the internationally accepted population ratio and its nursing employment capacity. While The Organisation for Economic Co-operation and Development (OECD) countries maintain an average of 8.8 nurses per 1000 people, Turkey lags behind with only 2.8 nurses per 1000 people, ranking second to last among OECD countries [25]. In an attempt to address the shortage of nurses in health services, overtime work and shifts are increasingly utilized, contributing to challenging working conditions in the healthcare environment in Turkey. These unfavorable conditions are identified as a significant factor leading to a projected surge in the number of nurses migrating abroad for better working conditions in the near future [26]. The existing shortage of nurses compels healthcare professionals to work extended hours, potentially driving nurses to consider leaving the country [26, 27]. A substantial proportion of nurses in Turkey are contemplating working in other countries [23, 26]. This inclination is intensified by changes in the nursing and health systems, coupled with financial challenges exacerbated by the COVID-19 pandemic [28]. Recognizing nurses as indispensable elements of a country’s health system, the shortage of nursing professionals emerges as a critical concern for ministries of health. While efforts to improve this situation are imperative, the risk of losing existing nurses due to brain drain poses a serious threat to effective health delivery in the country [23].

In the literature, no study has been identified that investigates the happiness, hope, and future expectations of nurses in Turkey. Hence, this study aims to determine the overall happiness, hope, and future expectations of nurses in Turkey in the year 2023, along with the factors influencing these aspects.

## Materials and methods

### Study type, population, and sample selection

This descriptive study was conducted on the population of all nurses providing nursing services in Turkey. Non-probability sampling method was used in the research. The research was completed with the participation of a total of 326 nurses from 52 provinces of Turkey.

### Data collection instruments

As data collection instruments, the Demographic Characteristics Form and the General Life Satisfaction Survey of the Turkish Statistical Institute (TÜİK) were utilized to assess the levels of overall happiness, hope, and future expectations.

#### Demographic characteristics form

This form, developed by the researchers, consisted of nine questions (age, gender, marital status, education level, province of employment, professional work experience, monthly income level, engagement in additional income-generating activities, and desire to work abroad) to determine the demographic characteristics of the participating nurses.

#### TUIK general life satisfaction survey

For this study, 14 questions from the TÜİK General Life Satisfaction Survey were used to assess the levels of overall happiness, hope, and future expectations. Of these questions, 1 is about overall happiness, 1 is about overall hope, 5 is about future expectations, 2 is about income satisfaction and the level of income to meet needs, 2 is about people and values that are sources of happiness, 1 is about personal development, 1 is about social reputation and 1 is about the working environment. The questions measuring overall happiness and overall hope were rated on a 5-point Likert scale, while the questions related to future expectations were rated on a 4-point Likert scale. The average Likert scale scores for overall happiness and hope were categorized as low if ¯X < 2.5, moderate if 2.5 < ¯X < 3.5, and high if ¯X > 3.5. Similarly, the average Likert scale scores for future expectations were categorized as low if ¯X < 1, moderate if 1 < ¯X < 2, and high if ¯X > 2 [29].

### Data collection

The study data was collected through an e-survey prepared using Google Forms and gathered in accordance with the principle of voluntary participation. The survey link of the research was conveyed through various social media groups, mostly composed of nurses working in different provinces. The initial section of the e-survey included a message informing participants about the study’s purpose, the approximate time required to complete the survey (approximately 5 min), the voluntary nature of participation, the option for participants to withdraw at any time, and the assurance of confidentiality for all personal information. After the initial notification, participating nurses proceeded with the e-survey, marked the informed consent section indicating their willingness to participate, and answered the survey questions. Data collection took place between July 11, 2023, and July 21, 2023. The inclusion criteria for the study were defined as being employed as a nurse in any healthcare institution and consenting to participate in the study.

### Ethical considerations

Prior to commencing the study, ethical approval was obtained from a state university’s Scientific Research and Publication Ethics Committee (dated July 10, 2023, with reference number 2023/70 − 01). It has been promised to the nurses that the information they give will be kept confidential and that this information will not be used anywhere other than the results of the research. Informed consent was obtained from the participating nurses. The research was conducted in accordance with the principles of the Helsinki Declaration.

### Analysis of study data

The study data were analyzed using the Statistical Package for the Social Sciences (SPSS) version 26.0 analysis software, and a statistical significance level of *p* < 0.05 was considered. The normal distribution of the data was assessed through the Shapiro-Wilk and Kolmogorov-Smirnov tests, as well as by examining Skewness and Kurtosis values. In the analysis of the data; numbers, percentages, arithmetic means, standard deviations, ANOVA, and independent sample t-test values were calculated.

## Results

When examining the demographic characteristics of the participating nurses in the study, it was found that 76.4% of the nurses were female, 27.0% were in the age range of 26–30, 58.9% were married, 68.2% had a bachelor’s degree, and 32.8% had a professional work experience of 1–5 years. In terms of monthly income, 70.6% of the nurses fell within the range of 15,000 to 19,999 TL, 92.9% did not engage in any additional income-generating activities, and 77.6% expressed a desire to practice nursing abroad if given the opportunity (Table 1).

**Table 1:** Demographic characteristics of participating nurses in the study

Features	n (326)	(%)
**Gender**		
Female Male	249 77	76.4 23.6
**Age**		
21–25 years old 26–30 years old 31–35 years old 36–40 years old 41–45 years old 46 years old and over	57 88 44 64 39 34	17.5 27.0 13.5 19.6 12.0 10.4
**Marital Status**		
Married Single	192 134	58.9 41.1
**Education Level**		
Health Vocational High School Associate degree Bachelor’s degree Master’s degree PhD	19 29 222 49 7	5.8 8.9 68.2 15.0 2.1
**Professional Working Time**		
1–5 years 6–10 years 11–15 years 16–20 years 21–25 years 26 years and above	107 66 61 46 20 26	32.8 20.3 18.7 14.1 6.1 8.0
**Monthly Income Level**		
Less than 10,000 TL 10,000–14,999 TL 15,000–19,999 TL 20,000–24,999 TL 25,000 TL and more	12 18 230 52 14	3.7 5.4 70.6 16.0 4.3
**Income-generating additional job status**		
Yes No	23 303	7.1 92.9
**Willingness to Practice Nursing Abroad**		
Yes No	253 73	77.6 22.4

When examining the opinions of the participating nurses regarding income satisfaction, sources of happiness, personal development, and working environments, it was found that 49.7% of the nurses were not satisfied with their income at all, and 40.8% mentioned that their income did not meet their needs. Of the nurses, 65.6% reported that the family members of the people who are the most important source of happiness, 55.2% reported that the most important source of happiness was health, and 37.4% reported that the most important feature that gives prestige in the society is moral characteristics. Additionally, 42.6% of the nurses stated that their personal development had increased in the last five years, and 34.0% mentioned that the most significant problem they encountered in their working environment was the disparity in salaries among professions (Table 2).

**Table 2:** Nurses’ opinions on income satisfaction, sources of happiness, personal development, and working environments

Features	n (326)	(%)
**Satisfaction with income**		
Not Satisfied at All Not Satisfied Moderate Satisfied	162 87 68 9	49.7 26.7 20.8 2.8
**Satisfaction of income to meet needs**		
Does Not Meet at All Does not meet Moderately meets Meets	97 133 84 12	29.8 40.8 25.7 3.7
**The most important person who is a source of happiness**		
All my family members My children Myself My mate Mum and Dad My friends Other	214 52 32 12 9 3 4	65.6 16.0 9.8 3.7 2.8 0.9 1.2
**The most important value that is a source of happiness**		
Health Love Money Success Work Other	180 74 37 19 4 12	55.2 22.7 11.3 5.8 1.2 3.8
**The most important feature that you think gives you a reputation in the society**		
Moral characteristic Decent family life Occupation/work performed Education Money Social environment Other	122 76 45 26 24 18 15	37.4 23.3 13.8 8.0 7.4 5.5 4.6
**Evaluation of personal development (last five years)**		
Developed Remained the same Regressed No opinion	139 60 113 14	42.6 18.4 34.7 4.3
**The most important problem you face in your work environment**		
Wage gap between professions Lack of merit Low amount of wages Working conditions Administrative problems Favouritism Other	111 62 44 40 30 30 9	34.0 19.0 13.5 12.3 9.2 9.2 2.8

The average scores for overall happiness, hope, and future expectations among the participating nurses were determined as 2.34 ± 0.98, 2.22 ± 0.95, and 1.26 ± 0.54, respectively. According to these findings, it was observed that the nurses had low levels of overall happiness and hope, while their future expectations were at a moderate level.

Based on the study data, a statistically significant relationship was identified between the nurses’ overall happiness level and variables such as marital status, desire to practice nursing abroad, satisfaction with income, income’s ability to meet needs, evaluation of the most important value contributing to happiness, and assessment of personal development (*p* < 0.05, Table 3). Accordingly, it was determined that nurses who were unmarried, aspired to work abroad, expressed dissatisfaction with their income, had income inadequacy to meet their needs, considered money as the primary source of happiness, and reported a decline in personal development over the last five years, had lower levels of overall happiness.

In the study, a significant relationship was found between nurses’ overall hope level and variables such as engaging in additional income-generating activities, desire to practice nursing abroad, satisfaction with income, income’s ability to meet needs, evaluation of the most important value contributing to happiness, and assessment of personal development (*p* < 0.05, Table 3). It was determined that nurses who engaged in additional income-generating activities, aspired to work abroad, expressed dissatisfaction with their income, had income inadequacy to meet their needs, considered money as the primary source of happiness, and reported a decline in personal development over the last five years had lower levels of overall hope.

Furthermore, the study revealed a statistically significant relationship between nurses’ future expectation levels and variables such as age, satisfaction with income, income’s ability to meet needs, and assessment of personal development (*p* < 0.05, Table 3). In this regard, it was determined that nurses between the ages of 41–45, who are satisfied with their income, whose income meets their needs, and whose personal development has increased in the last five years, have a higher level of future expectation.

**Table 3:** Variables associated with nurses’ overall happiness, hope, and future expectation levels

Features	n (326)	Overall Happiness ($\bar x \pm ss$±SS)	Overall Hope ($\bar x \pm ss$±SS)	Future Expectations ($\bar x \pm ss$±SS)	
$\bar x \pm ss$ ***±SS***		2.34 ± 0.98	2.22 ± 0.95	1.26 ± 0.54	
**Marital status**					
Married Single **Testing and Materiality**	192 134	2.45 ± 1.02 2.17 ± 0.90 t = 2.586,p*=0.010	2.29 ± 0.94 2.13 ± 0.96 t = 1.464, *p* = 0.144	1.26 ± 0.56 1.24 ± 0.45 t = 1.301, *p* = 0.072	
**Age**					
21–25 years old ^a^ 26–30 years old ^b^ 31–35 years old ^c^ 36–40 years old ^d^ 41–45 years old ^e^ 46 years old and over ^f^ **Test and Materiality**	57 88 44 64 39 34	2.24 ± 0.87 2.29 ± 1.04 2.22 ± 1.05 2.48 ± 1.00 2.48 ± 1.07 2.35 ± 0.81 F = 0.697,*p* = 0.626	2.19 ± 0.89 2.18 ± 1.01 2.25 ± 0.96 2.23 ± 0.95 2.28 ± 0.91 2.29 ± 1.00 F = 0.111,*p* = 0.989	1.24 ± 0.48 1.15 ± 0.48 1.30 ± 0.48 1.19 ± 0.49 1.45 ± 0.73 1.40 ± 0.62 F = 2.456,p*=0.033 **Difference; e > b**	
**Income generating additional work**					
Yes No **Test and Materiality**	23 303	2.08 ± 1.04 2.36 ± 0.92 t=-1.231, *p* = 0.230	1.86 ± 0.81 2.25 ± 0.96 t=-2.152, p*=0.041	1.23 ± 0.36 1.26 ± 0.55 t=-1.346, *p* = 0.073	
**Desire to practice nursing abroad**					
Yes No **Test and Materiality**	253 73	2.23 ± 0.93 2.71 ± 1.07 t=-3.424,p**=0.001	2.13 ± 0.90 2.53 ± 1.05 t=-3.159,p*=0.002	1.23 ± 0.48 1.33 ± 0.71 t=-1.350,*p* = 0.178	
**Satisfaction with income**					
Not Satisfied at all ^a^ Not Satisfied ^b^ Moderate ^c^ I am satisfied ^d^ **Test and Materiality**	162 87 68 9	1.91 ± 0.88 2.52 ± 0.83 2.94 ± 0.89 3.77 ± 0.66 F = 3.619,p**=0.000 **Difference; d > c, b, a**	1.85 ± 0.87 2.41 ± 0.88 2.67 ± 0.80 3.66 ± 0.86 F = 5.737,p**=0.000 **Difference; a < b, c, d**	1.16 ± 0.35 1.33 ± 0.64 1.30 ± 0.66 1.97 ± 0.65 F = 7.920,p**=0.000 **Difference; d > c, b, a**	
**Satisfaction of income to meet needs**					
Does Not Meet at All ^a^ Does not meet ^b^ Moderately Meets ^c^ Meets ^d^ **Test and Materiality**	97 133 84 12	1.73 ± 0.82 2.33 ± 0.87 2.89 ± 0.89 3.58 ± 0.66 F = 6.208,p**=0.000 **Difference; d > a, b, c**	1.74 ± 0.84 2.21 ± 0.89 2.71 ± 0.92 2.83 ± 0.71 F = 2.244,p**=0.000 **Difference; d > a, b, c**	1.10 ± 0.38 1.27 ± 0.51 1.35 ± 0.68 1.71 ± 0.56 F = 6.690,p**=0.000 **Difference; d > a, c > a**	
**The most important value that is a source of happiness**					
Health ^a^ Love ^b^ Money ^c^ Success ^d^ Work ^e^ Other ^f^ **Test and Materiality**	180 74 37 19 4 12	2.44 ± 098 2.55 ± 0.96 1.56 ± 0.64 2.21 ± 0.97 2.00 ± 0.81 2.27 ± 1.10 F = 5.217,p**=0.000 **Difference; c < a, b**	2.31 ± 0.88 2.37 ± 1.02 1.56 ± 0.80 2.10 ± 1.04 1.75 ± 0.95 2.45 ± 1.12 F = 4.355,p**=0.000 **Difference; c < a, b**	1.26 ± 0.58 1.32 ± 0.57 1.15 ± 0.35 1.13 ± 0.36 1.25 ± 0.37 1.26 ± 0.49 F = 0.692, *p* = 0.630	
**Evaluation of personal development (last five years)**					
Developed ^a^ Stayed the same ^b^ Regressed ^c^ No idea ^d^ **Test and Materiality**	139 60 113 14	2.74 ± 0.98 2.31 ± 0.81 1.90 ± 0.89 2.07 ± 0.82 F = 7.725,p**=0.000 **Difference; c < a, b**	2.61 ± 1.03 2.13 ± 0.76 1.79 ± 0.75 2.28 ± 0.72 F = 7.700,p**=0.000 **Difference; c < a, b**	1.43 ± 0.63 1.21 ± 0.43 1.11 ± 0.39 0.90 ± 0.47 F = 10.662,p**=0.000 **Difference; a > b, c, d**	

t = independent-Samples t Test, F = ANOVA, *p* = statistical significance level, *p < 0.05, **p < 0.001. According to the results of the multiple comparison test (posthoc-test: Tukey), different letters indicated by alphabetical superscripts (a,b,c,d,e,f) indicate that there is a significant difference between the scale scores

## Discussion

In the United Nations World Happiness Report, it has been reported that in the year 2023, Turkey ranked 106th out of 137 countries [30]. According to the results of the Life Satisfaction Survey conducted annually by the Turkish Statistical Institute (TUIK) since 2003, half of the adult population in Turkey claimed to be happy in the year 2022 [31]. In this study, we concluded that the overall happiness levels of nurses are low. In the literature, studies conducted in various countries regarding the happiness levels of nurses have indicated that their levels are either low or modera [6, 16, 17]. While our study results are consistent with the literature in this aspect, it is a fact that the happiness levels of nurses in Turkey are lower than those of the general population. This situation could be attributed to various factors, including the working conditions of nurses, the extraordinary circumstances under which nurses worked during the COVID-19 pandemic, as well as the economic and political factors within the country because the concept of happiness is economically related to inflation, employment, national income, individual income and consumption, social security and social policies [3].

It has been reported that the happiness of nurses is affected by various factors such as gender, age, professional working years, marital status, physical health status, quality of life, reason for choosing nursing, salary satisfaction, workload, patient profile, interpersonal relationships, cognitive flexibility, and emotion management skills [15, 16, 18, 19, 32, 33]. In this study, it was concluded that happiness in nurses was not affected by age, gender, years of employment, educational level, or monthly income level, but it was related to marital status, doing additional income-generating work, desire to work as a nurse abroad, satisfaction with income, the ability of income to meet needs, the value that is the most important source of happiness (health, love, and money), and the level of personal development in the last five years. Studies have shown that there is a positive relationship between economic status and happiness and that salary satisfaction is an important predictor of nurses’ happiness [18]. Nearly half of the nurses participating in the study stated that they were not satisfied with their income at all and that their income was not enough to meet their needs, while the majority of the nurses reported that the most important problem they faced in their working environment was the wage gap between occupations. In this regard, our study’s findings align with those of the existing literature.

All the nurses involved in this study are employed in public hospitals. The preference for working in public hospitals is attributed to the relatively superior working conditions and salaries offered in comparison to private hospitals in Turkey. Despite these advantages, a notable majority of nurses express dissatisfaction with the current level of income, considering it insufficient to meet their needs adequately. The study suggests that extending the research scope to include nurses working in private hospitals would provide valuable insights.

It was found that three out of every four nurses who participated in our study wanted to work abroad and nurses who wanted to work abroad had lower levels of happiness and hope. We believe that this finding is significant for two possible reasons. The first is that unhappiness and hopelessness may lead nurses to think of working in another country, and the second is that the nurses’ desire to work abroad may cause the need for qualified nurses in the future in Turkey. In the study conducted by THD, 76.3% of nurses wanted to practice nursing abroad, while 23.7% reported that they did not want to practice nursing in Turkey [26]. It has been reported that the worldwide demand for nurses will exceed 7.6 million by 2030, the nurse shortage will become a global problem, and the COVID-19 pandemic has exacerbated the nurse shortage [10, 34]. The COVID-19 pandemic has aggravated the challenges within the nursing profession, creating a dissonance between ideal expectations and the realities faced in practice, leading to emotional burnout. Prolonged work hours amid high stress and uncertainty during the pandemic, coupled with factors such as changing nursing unit locations and increased workloads, have been observed to accelerate the experience of burnout among nurses [28]. The adverse working conditions during the pandemic have, in turn, increased nurses’ intentions to migrate to developed countries where they anticipate finding more ideal and supportive working environments [27]. In recent years, there has been an increase in the migration of nurses worldwide, driven by factors such as political and economic reasons, concerns about the future, employment issues, and the desire for professional development [35, 36]. In underdeveloped countries, nurses often face multiple factors that drive them toward voluntary migration, like insufficient respect for the nursing profession, low wages relative to the work performed, limited participation in social life and educational opportunities, and economic instability [21]. Our study identified similar factors influencing nurse migration in Turkey.

To address the challenges of today and the future, it is important to understand the past. At a time when the global climate crisis is affecting the entire world and poses a significant threat to the future, nurses play a crucial role in preparing communities for the increasing frequency and impact of disasters, similar to their role during the COVID-19 pandemic [37]. Nurses play essential roles in public health, clinical care, emergency response, research, and advocacy to mitigate and respond to the health consequences of the global climate crisis [38]. As one of the most trusted professions globally, nurses are at the forefront of the healthcare system, bearing the cost of increasing disease prevalence and more frequent disaster events [38, 39]. Climate change is already affecting human health and will have an even greater impact in the future [38]. Therefore, considering the existing shortage of nurses in Turkey and the anticipated global demand for nurses in the future, improving working conditions, income levels, and supporting sources of happiness and hope for nurses should be considered by leaders and policymakers to prevent qualified nurse migration from Turkey.

Happiness not only brings a sense of well-being to individuals but also leads to optimism and hopeful attitudes toward the future, enabling innovative and productive choices [15]. When nurses perceive fair treatment, they tend to have more trust in their work and their supervisors, thus working with hope and motivation [19]. Hope is influenced by various factors such as workload, working conditions, team relationships, management style, and rewards [10]. The study concluded that nurses have low levels of overall hope and that nearly all variables affecting happiness also influence hope. The hopeful outlook of nurses not only affects their well-being but also impacts the hope levels of patients and their families [40]. Therefore, the low levels of hope among nurses in Turkey could negatively affect nurse-patient relationships and the delivery of quality healthcare services.

Expectations for the future encompass individuals’ plans, desires, and fears concerning various aspects of life in the near or distant future. There is a positive relationship between hope, happiness, and future expectations [8, 12, 13]. The study showed that the level of future expectations among nurses is moderate. Positive future expectations are related not only to an individual’s plans and goals for the future but also to their social relationships and household income [1]. In the study, it was determined that nurses aged 26–30, those who were either dissatisfied or moderately satisfied with their income, those whose income did not meet their needs, and those whose personal development had decreased or remained the same in the past five years, had lower levels of overall future expectations. These findings suggest that young nurses, especially those who have recently entered the nursing profession, are at risk in terms of continuing in this profession and maintaining their mental well-being. Therefore, supporting the personal development of newly established young nurses and ensuring that they achieve an income level that meets their needs will contribute to an increase in their future expectations.

## Limitations

Nurses working in hospitals affiliated with the Ministry of Health in 52 different provinces of Turkey participated in the research. Although this increases the generalizability of the research, the results of the research cannot be generalized to all nurses in Turkey. In addition since participation in the research is voluntary, the results only reflect the opinions of the nurses who participated voluntarily. Additionally, collecting research data using a self-report survey may have biased the findings.

## Conclusion

In this study, it was determined that nurses in Turkey have low levels of overall happiness and hope, and moderate levels of future expectations. According to the study findings, the happiness and hope levels of nurses were found to be related to variables such as engaging in supplementary income-generating work, desire to work as a nurse abroad, satisfaction with income, income sufficiency, the most important source of happiness (health, love, and money), and personal development level over the past five years. It was also established that nurses’ future expectations are related to age, satisfaction with income, income sufficiency, and personal development level over the past five years. In line with these results, it is recommended to increase the income level of nurses in Turkey at a level that can satisfy them and meet their needs, to create social, cultural, and educational opportunities that can contribute to the personal development of nurses, to make strategic and political arrangements that can increase the level of happiness, hope and expectation for the future of nurses and reduce the thought of migration abroad.

## Data Availability

The datasets used and analyzed during this study are available from the corresponding author on reasonable request.
